# Diagnostic accuracy of a rapid nucleic acid test for group A streptococcal pharyngitis using saliva samples: protocol for a prospective multicenter study in primary care

**DOI:** 10.1186/s41512-023-00150-4

**Published:** 2023-07-13

**Authors:** Robert Touitou, Philippe Bidet, Constance Dubois, Henri Partouche, Stéphane Bonacorsi, Camille Jung, Robert Cohen, Corinne Levy, Jérémie F. Cohen

**Affiliations:** 1 Association Enseignement Formation Généralistes Hospitaliers — Croix Saint Simon, Paris, France; 2 Department of Microbiology, Robert Debré Hospital, AP-HP, Université Paris Cité, Paris, France; 3 Centre of Research in Epidemiology and Statistics (Inserm UMR 1153), Université Paris Cité, Paris, France; 4 Department of General Practice, Université Paris Cité, Paris, France; 5 Clinical Research Centre, Centre Hospitalier Intercommunal de Créteil, Créteil, France; 6 Association Clinique Et Thérapeutique Infantile du Val-de-Marne (ACTIV), Créteil, France; 7 Department of General Pediatrics and Pediatric Infectious Diseases, Hôpital Necker-Enfants Malades, AP-HP, Université Paris Cité, Paris, France

**Keywords:** Acute pharyngitis, Sore throat, Rapid antigen detection tests, Rapid nucleic acid tests, Group A streptococcus, Primary care, Saliva, Diagnostic accuracy, Sensitivity, Specificity

## Abstract

**Background:**

Group A streptococcus is found in 20–40% of cases of childhood pharyngitis; the remaining cases are viral. Streptococcal pharyngitis (“strep throat”) is usually treated with antibiotics, while these are not indicated in viral cases. Most guidelines recommend relying on a diagnostic test confirming the presence of group A streptococcus before prescribing antibiotics. Conventional first-line tests are rapid antigen detection tests based on throat swabs. Recently, rapid nucleic acid tests were developed; they allow the detection of elements of the genome of group A streptococcus. We hypothesize that these rapid nucleic acid tests are sensitive enough to be performed on saliva samples instead of throat swabs, which could be more convenient in practice.

**Methods:**

This is a multicenter, prospective diagnostic accuracy study evaluating the performance of a rapid nucleic acid test for group A streptococcus (Abbott ID NOW STREP A2) in saliva, compared with a conventional pharyngeal rapid antigen detection test (EXACTO PRO STREPTATEST, lateral flow assay, comparator test), with a composite reference standard of throat culture and group A streptococcus PCR in children with pharyngitis in primary care (i.e., 27 primary care pediatricians or general practitioners). To ensure group A streptococcus is not missed, the salivary rapid nucleic acid test requires a minimally acceptable value of sensitivity (primary outcome) set at 80%. Assuming 35% of participants will have group A streptococcus, we will recruit 800 consecutive children with pharyngitis. Secondary outcomes will include difference in sensitivity between the pharyngeal rapid antigen detection test and the salivary rapid nucleic acid test; variability in sensitivity and specificity of the salivary rapid nucleic acid test with the level of McIsaac score; time to obtain the result of the salivary rapid nucleic acid test; patient, physician, and parents satisfaction; and barriers and facilitators to using rapid tests for group A streptococcus in primary care.

**Ethics and dissemination:**

Approved by the Institutional Review Board “Comité de protection des personnes Ile de France I” (no. 2022-A00085-38). Results will be presented at international meetings and disseminated in peer-reviewed journals.

**Trial registration number:**

ClinicalTrials.gov: NCT05521568.

## Introduction

Acute pharyngitis is responsible for 15 million visits to physicians each year in the USA [1]. Group A streptococcus is found in 20 to 40% of cases of childhood pharyngitis (37% in a recent meta-analysis) [2]; the remaining cases are viral. Streptococcal pharyngitis (“strep throat”) is usually treated with antibiotics to accelerate symptom relief, prevent complications, and reduce the spread of group A streptococcus [3, 4], while antibiotics are not indicated in viral cases. Because signs and symptoms of streptococcal and viral cases overlap, diagnosing streptococcal pharyngitis on clinical grounds is considered unreliable, even for expert clinicians, and most pediatric guidelines recommend relying on a diagnostic test confirming the presence of group A streptococcus to select who should receive antibiotics [1, 5]. The usual reference standard test for diagnosing streptococcal pharyngitis is a throat culture in the microbiology laboratory using a throat swab. However, throat culture is impractical for routine care because the result is only available after 24–48 h [6].

Rapid antigen detection tests have been developed since the 1980s. They provide a result within minutes and can be performed at the point of care. Several studies and meta-analyses have evaluated rapid antigen detection tests in children with pharyngitis and shown that they allow diagnosis with high sensitivity (86% on average) and high specificity (95% on average) [7], which is better than clinical scoring systems (e.g., Centor and McIsaac scores) [8]. In many countries, recommendations are to prescribe antibiotics only with a positive rapid antigen detection test result. Several randomized trials and meta-analyses have shown that implementing rapid antigen detection tests significantly reduces antibiotic prescriptions by 25 percentage points on average [9]. Despite their acceptable diagnostic accuracy, substantial impact on antibiotic use, and low cost (i.e., about US $1/test), there is still a failure in implementing rapid antigen detection tests. For example, according to the latest study in primary care in France, including more than 200,000 general medicine consultations, the antibiotic prescriptions rate in French children with pharyngitis in general practice was 67% [10]. The main reasons cited by primary care physicians for not performing rapid antigen detection tests are lack of time and difficulty in performing them. One of the main limitations of current rapid antigen detection tests, particularly in children, is the need for a throat swab. In a recent evaluation, the sensitivity of a rapid antigen detection test varied from 56 to 96% among physicians, and this was likely due to a suboptimal throat swab technique [11]. Another limitation of pharyngeal rapid antigen detection tests is that pharyngeal swabbing may cause the patient to cough, potentially contaminating the people and the environment in the room.

Since the 2010s, rapid nucleic acid tests for group A streptococcus that use polymerase chain reaction (PCR) and other nucleic acid amplification techniques have been available [12]. They have the advantage of being very sensitive and able to give a result within minutes. Some of these tests seem simple, compact, and quick enough to be implemented in primary care [13]. A recent meta-analysis showed that these rapid nucleic acid tests for group A streptococcus have a sensitivity of 97.5% with a specificity of 95.1% on average [14]. A North-American pilot study conducted on 20 patients showed that a rapid nucleic acid test for group A streptococcus (Roche’s cobas Liat test) could be performed on saliva with satisfactory performance and results highly consistent with those of a conventional pharyngeal rapid antigen detection test [15]. Replacing conventional pharyngeal rapid antigen detection tests with less invasive salivary rapid nucleic acid tests would allow for keeping the benefits of rapid tests while avoiding the throat swab step.

Here, we will carry out the first large-scale study to evaluate the diagnostic accuracy of a rapid nucleic acid test for group A streptococcus on saliva in children with pharyngitis in primary care. We will also compare the accuracy of the salivary rapid nucleic acid test to that of the rapid antigen detection test that is currently used in routine care.

## Materials and methods

### Study objectives

The primary objective of this study is to evaluate the sensitivity of a rapid nucleic acid test for group A streptococcus on saliva in children in primary care.

Secondary objective(s) include the following:

- To compare the diagnostic accuracy of the salivary rapid nucleic acid test versus that of a conventional pharyngeal rapid antigen detection test (EXACTO PRO STREPTATEST, a lateral flow assay)
- To evaluate the variability in diagnostic accuracy of the salivary rapid nucleic acid test according to the clinical severity of patients (“spectrum effect”)
- To evaluate the barriers and facilitators to using rapid tests for group A streptococcus in primary care
- To evaluate the satisfaction of physicians and users (children and their parents)

### Study design

This is an observational, prospective, multicenter, cross-sectional, comparative, single-gate, double-blinded, manufacturer-independent diagnostic accuracy study.

### Study setting

The study will be conducted in 27 private practices of general practitioners and pediatricians in France. If necessary, further practices may be included to ensure recruitment within the intended time frame.

### Eligibility criteria

#### Inclusion criteria

- Children 3–15 years
- Managed in primary care by a general practitioner or primary care pediatrician
- With a clinical diagnosis of acute pharyngitis, defined as an inflammation of the pharynx and/or tonsils (i.e., erythema with or without exudate) or acute sore throat (even if without local signs of pharyngeal inflammation)
- Non-opposition of the accompanying parent(s)

#### Exclusion criteria

- Children who received antibiotics within 7 days before potential inclusion
- Children already enrolled in the study for the same episode of pharyngitis

### Recruitment

Children with acute pharyngitis will be recruited consecutively from 27 primary care private practices throughout France over a period of 18 months. Recruitment is expected to start in the first trimester of 2023.

### Data collection

Data collection procedures and timing are described in Table 1. First, physicians will collect sociodemographic and clinical data. Second, they will perform the salivary rapid nucleic acid test (index test). Third, they will take a throat sample for the reference standard and the comparator test.

**Table 1:** Data collection procedures and timing

Type of data	Inclusion visit	One-month follow-up phone call	After the end of patient inclusions
Baseline information			
Non-opposition to participate in the study	X		
Sociodemographic data	X		
Clinical data (including McIsaac criteria)	X		
Biospecimens			
Saliva sample (for the salivary rapid nucleic acid test)	X		
Double throat swab (one for the pharyngeal rapid antigen detection test, one for the composite reference standard)	X		
Test results			
Result of the salivary rapid nucleic acid test (index test)^a^	X		
Result of the pharyngeal rapid antigen detection test (comparator test)^a^	X		
Result of group A streptococcus culture and group A streptococcus PCR (reference standard)^b^	X		
Satisfaction			
Patient satisfaction (Likert)	X		
Physician satisfaction (Likert)	X		
Parent satisfaction (Likert)	X		
Antibiotic prescription	X		
Clinical evolution (i.e., whether the episode of pharyngitis healed, worsened, or relapsed)		X	
Qualitative survey for participating physicians (i.e., barriers and facilitators regarding the use of rapid tests)			X^c^

*PCR* Polymerase chain reaction

^a^Performed at the point of care

^b^Performed in the microbiology laboratory

^c^Each participating physician will take the survey once

### Clinical data and data storage

For each participant, the following information will be collected using an online case report form (eCRF):

- Patient age
- McIsaac score criteria
- Result of the salivary rapid nucleic acid test (saliva swab)
- Result of the pharyngeal rapid antigen detection test (throat swab)
- Time to obtain the result of the rapid nucleic acid test (measured by the clinician in charge, using a chronometer, a watch, or a smartphone)
- Antibiotic prescription (yes/no and details if antibiotics are prescribed)
- Patient satisfaction (Likert scale) regarding the pharyngeal rapid antigen detection test and the salivary rapid nucleic acid test
- Physician satisfaction (Likert scale) regarding the pharyngeal rapid antigen detection test and the salivary rapid nucleic acid test
- Parent satisfaction (Likert scale) regarding the pharyngeal rapid antigen detection test and the salivary rapid nucleic acid test
- One-month follow-up phone call (whether the episode healed, worsened, or relapsed)

Anonymized data will be stored on a secured server for 15 years after the completion of the study.

### Index test

**Fig. 1:**
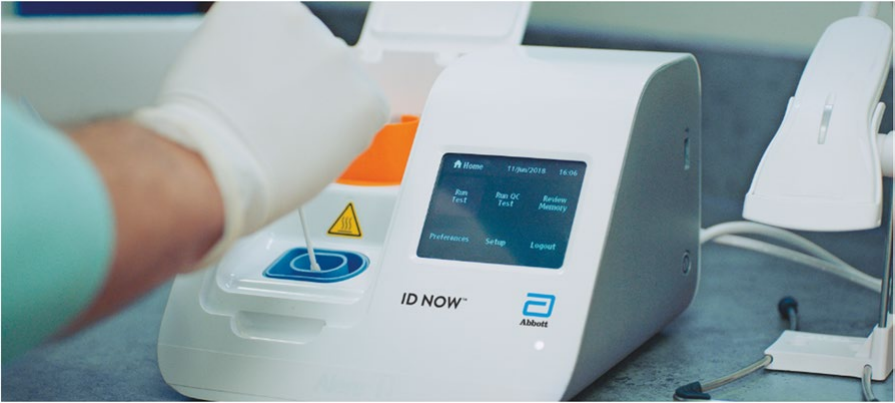
Abbott’s ID NOW STREP A 2 (rapid nucleic acid test)

The test under evaluation (“index test”) is the rapid nucleic acid test for group A streptococcus Abbott ID NOW STREP A 2 (formerly known as Alere i strep A test; Fig. 1), performed on saliva samples instead of throat swabs. This rapid nucleic acid test was chosen because of its ease of use and several previous evaluations showing high diagnostic accuracy on throat swabs [16–18]. The ID NOW STREP A 2 does not rely on a rapid PCR technique but is based on the principle of isothermal nicking enzyme amplification reaction. It allows rapid detection of group A streptococcus nucleic acids by amplifying a sequence of the *cepA* gene, which encodes the C5a peptidase, an important streptococcal virulence factor. The swab to be tested is directly inserted into the platform for 10 s, without any complex steps. The ID NOW STREP A 2 test gives a binary result after 2 to 6 min. The rapid nucleic acid test will be performed at the point of care by participating primary care practitioners, using salivary swabs instead of throat swabs. Saliva samples will be collected using the swabs provided by Abbott in the test kits. Children will be invited to suck on the swab for 30 s as they would for a lollipop. In a preliminary study, we tested the swab with saliva from asymptomatic volunteers and found no inhibitor; the test also proved able to detect group A streptococcus when testing three strains of group A streptococcus with progressive dilutions (see Appendix 1). Rapid nucleic acid test results will not be used for patient management. Clinicians performing the saliva rapid nucleic acid test will be blinded to the result of the reference standard and the result of the pharyngeal rapid antigen detection test but not to clinical information. Participating practitioners will receive specific training before the study.

### Reference standard

The reference standard will be a composite of culture and group A streptococcus PCR, using a throat swab. Throat culture is the most accepted reference standard for diagnosing strep throat. However, studies have shown that culture may be falsely negative in the presence of other bacteria, such as *Staphylococcus aureus*, and in such cases, group A streptococcus PCR would be positive elsewhere [19]. Throat samples will be obtained by use of a double-swab collection-transportation system (COPAN Diagnostics, Corona, CA, USA). Swab no. 1 will be used immediately to perform the pharyngeal rapid antigen detection test (see below), as per usual care. Swab no. 2 will be put in the Amies agar transportation system, held at ambient temperature, and sent within 72 h to the Robert Debre Hospital microbiology laboratory by an express messenger service. Upon reception, throat swabs will be used to perform throat cultures using standard techniques, as described elsewhere [20]. Swab no. 2 will also be used to perform a group-A-streptococcus-specific PCR, as described elsewhere [19]. Samples with a positive culture and/or a positive PCR test result will be classified as group A streptococcus positives. Samples with a negative culture and a negative PCR will be classified as group A streptococcus negatives. Executors of the reference standard will be blinded to clinical data, including the result of the pharyngeal rapid antigen detection test and salivary rapid nucleic acid test.

### Comparator test

As per usual care, all children will undergo a conventional pharyngeal rapid antigen detection test, using swab no. 1 (see above). This lateral flow assay is the one used in France and is the only commercial kit that is officially recommended by the National Health Insurance system. It detects Lancefield’s group A antigen, which is specific to group A streptococcus and provides a binary result. The test will be performed at the point of care by primary care practitioners participating in the study, as per usual care. Clinicians performing the rapid antigen detection test will not be blinded to clinical information and results of the salivary rapid nucleic acid test.

### Biospecimen storage

Double throat swabs will be taken from each participant. One will be used during consultation to perform the pharyngeal rapid antigen detection test. One will be sent to the microbiology laboratory to perform the reference standard test. If the throat swab used in the lab grows group A streptococcus on culture, the strain will be stored at − 80 °C for 5 years.

### Outcome measures

#### Primary outcome measures

1. Sensitivity of the Abbott ID NOW STREP A 2 rapid nucleic acid test on saliva samples.

#### Secondary outcome measures

1. Difference in sensitivity between the salivary rapid nucleic acid test (Abbott ID NOW STREP A 2) and the classical pharyngeal rapid antigen detection test (EXACTO PRO STREPTATEST)
2. Other diagnostic accuracy measures (i.e., specificity and predictive values) of the Abbott ID NOW STREP A 2 rapid nucleic acid test on saliva samples
3. Variability in sensitivity and specificity of the salivary rapid nucleic acid test according to the McIsaac score (“spectrum effect”)
4. Time needed to perform the salivary rapid nucleic acid test and obtain the results
5. Satisfaction of the child, the physician, and the accompanying parent(s)
6. Barriers and facilitators to using rapid tests among primary care practitioners

### Statistical analysis

#### Descriptive analysis

The flow of patients through the study will be described in a flow chart.

Descriptive statistics will be carried out on all study participants and will include the following:

- For quantitative variables: Number of patients, mean, standard deviation, median, and interquartile range
- For categorical variables: Number of patients and proportion in each category
- 95% confidence intervals will be computed for all estimates.

Group comparisons will involve the following:

- For quantitative variables: Student *t*-test or Mann–Whitney *U*-test, as appropriate
- For categorical variables: Chi-square or Fisher’s exact test, as appropriate

#### Diagnostic accuracy

Diagnostic accuracy performance measures and their 95% confidence intervals will be computed based on the corresponding contingency tables, with a composite reference standard of culture and PCR. Contingency tables will be reported. We will calculate diagnostic accuracy measures (i.e., sensitivity, specificity, and predictive values) separately for the salivary rapid nucleic acid test and the pharyngeal rapid antigen detection test.

Then, we will compare the sensitivity and specificity of the salivary rapid nucleic acid test versus those of the conventional pharyngeal rapid antigen detection test using McNemar’s chi-square test for paired data. All study participants will undergo both tests (Abbott ID NOW STREP A 2 and EXACTO PRO STREPTATEST), but the the comparison will only include patients for whom the result of both tests is available.

#### Missing and indeterminate test results

In the principal diagnostic accuracy analysis, missing and indeterminate rapid nucleic acid test and reference standard results will be excluded from contingency tables and accuracy estimates. In sensitivity analyses, missing and indeterminate index tests will be considered as being rapid nucleic acid test positives (scenario 1) or rapid nucleic acid test negatives (scenario 2). Missing reference standard results will not be imputed; they will be excluded.

#### Analyses of variability in diagnostic accuracy

We will use the Cochran-Armitage chi-square trend test to evaluate the presence of a “spectrum effect.” For that purpose, we will test the hypothesis of an increase in sensitivity and a concomitant decrease in specificity when clinical severity (as measured by the McIsaac score) increases, as already shown for the rapid antigen detection test [20, 21].

The primary analysis will not account for the multicenter aspect of the study. However, in an additional analysis, we will explore the potential for variability in diagnostic accuracy across participating centers, e.g., through random-effects logistic modeling.

#### Analysis of patient, physician, and parent satisfaction

Patient, physician, and parent satisfaction data will be described using standard descriptive statistics (see above). We will then explore whether patient and physician satisfaction depends on patient and physician characteristics, respectively, using classic hypothesis tests (Student, Mann–Whitney, chi-square, Fisher’s exact test).

#### Analysis of barriers and facilitators to using rapid tests

At the end of the study period, all participating physicians will be invited to complete an online survey exploring their knowledge, attitudes, and practices regarding sore throat and the use of rapid tests, using a questionnaire adapted from previous similar studies [11, 22]. Survey results will be described and then analyzed in order to identify barriers and facilitators to using rapid antigen detection tests and rapid nucleic acid tests.

### Sample size calculation

Our main outcome measure is the sensitivity of Abbott’s ID NOW STREP A 2 on saliva samples. We based our sample size calculation on the objective of showing that the sensitivity of the salivary rapid nucleic acid test for group A streptococcus should reach at least 80% (expert opinion) [23]. We also hypothesized that the sensitivity of the salivary rapid nucleic acid test would be equal to that of the pharyngeal rapid antigen detection test [15].

Sample size was calculated in Stata/SE 15 (Statacorp, College Station, TX, USA) through a bilateral Wald test for comparing one proportion to a reference value (*power oneproportion* command) with the following settings:

- Minimally acceptable value of sensitivity of the salivary rapid nucleic acid test (i.e., p_0_): 80%
- Expected sensitivity of the salivary rapid nucleic acid test (i.e., p_a_): 86% [7]
- Expected prevalence of group A streptococcus: 35% [20]
- Alpha risk: 5%

With these assumptions, we would need to recruit 263 children with streptococcal pharyngitis, resulting in a total sample size of 751 children with pharyngitis. Assuming about 5% of children lost between inclusion and analysis, we will include a total of 800 children with acute pharyngitis.

### Assessment of possible adverse events

Saliva sampling is a safe and noninvasive technique. It is extremely unlikely that any adverse events could arise during saliva tests, but adverse events will be systematically collected. Standard clinical care with clinical management based on throat swabs will continue during the completion of the study. Any adverse events that could arise during pharyngeal tests will be collected.

### Ethics and dissemination

This study has been approved by the Institutional Review Board “Comité de protection des personnes Ile de France I” (no. 2022-A00085-38). The study was registered on ClinicalTrials.gov (NCT05521568). The protocol and full text of this study will be reported according to STARD 2015 [24]. Results will be presented at international meetings and disseminated in peer-reviewed journals.

## Data Availability

Plan to share individual participant data: undecided.

## Appendix 1

### Results of the preliminary study

In a preliminary study, we explored the presence of inhibitors and assessed the feasibility of reducing the collection time to 10 s. However, we concluded that maintaining a collection time of 30 s is the optimal choice, as detailed below.

- In the first experiment, saliva samples were collected from 16 healthy individuals ranging from 4 to 60 years of age. These samples were subjected to rapid molecular testing using different saliva collection durations, ranging from 10 to 30 s. All the tests yielded negative results, indicating the absence of inhibitors within the saliva samples.
- In the second experiment, saliva samples were obtained from 9 children who did not have pharyngitis. These samples were inoculated with controlled quantities of Group A Streptococcus, using strains known to be highly prevalent in childhood pharyngitis, namely M1, M4, and M89 serotypes. Each sample was inoculated with varying amounts of bacteria, approximately 1500, 150, and 15 colony-forming units (CFU)/mL, which corresponds to approximately 200, 20, and 2 CFU per swab, respectively. Rapid molecular tests were conducted for each sample. First, the swab was left in contact with saliva for 10 s; if the result was negative, a second test was performed with a prolonged contact time of 30 s. With an inoculum of 1500 CFU/ml, test results were consistently positive for all group A streptococcus strains after 10 s (100%, 9/9). With an inoculum of 150 CFU/ml, the test results were positive in 44% (4/9) of cases after 10 s, and extending the contact time to 30 s allowed the detection of one additional case. With an inoculum of 15 CFU/ml, test results were systematically negative after 10 s (0%, 0/9), and prolonging the contact time to 30 s did not lead to the detection of any additional case. We concluded that 30 s of saliva collection might provide improved sensitivity compared with 10 s.

## Abbreviations

eCRF: Electronic case report form
PCR: Polymerase chain reaction
